# Protective effect of Petroselinum crispum extract in abortion using prostadin-induced renal dysfunction in female rats

**Published:** 2014

**Authors:** Maryam Rezazad, Farah Farokhi

**Affiliations:** 1 *Department of Biology, Urmia University, Urmia, I.R. Iran*

**Keywords:** *Abortion*, *Dysfunction*, *Kidney*, *Petroselinumcrispum*

## Abstract

**Objective:** Present study investigated the effects of parsley extract on pregnant rat kidneys which have undergone clinical abortion using prostaglandins. The renal protective effect of parsley extract was evaluated in pregnant rats which had an abortion. Parsley was used due to its antioxidant properties.

**Materials and Methods:** Fifty-four female rats were divided in 9 groups of 6: control pregnant, two pregnant groups which received parsley extract and prostadin, two non-pregnant groups treated with parsley extract and prostadin, a group administered with both treatments, and three groups which received parsley extract in pre-implantation, implantation, and post-implantation periods of embryos. Ethanolic extract (5 mg/kg) was given daily to animals for 18 days of pregnancy period. Parameters such as malondialdehyde (MDA), total antioxidant statues (TAS), creatinine, and urea were measured using biochemical assays. Histopathologic studies were also done with Hematoxylin-Eosin staining method.

**Results:** After 18 days of treatment, significant differences were observed in serum creatinine, urea, and MDA and TAS levels. Kidney cross-sections showed edema in prostadin-treated rats while improvements in parsley + prostadin -treated rats were observed.

**Conclusion:** These results suggested that ethanolic extract of Petroselinum crispum reduced the dysfunction in rats kidney caused by prostadin-induced abortion and could have beneficial effect in reducing the progression of prostaglandin-induced edema.

## Introduction

Parsley (*Petroselinum crispum*) of the family Apiaceae, is a native of central Mediterranean, widely cultivated as an herb, spice, and vegetable. Its germination is slow, due to furanocoumarins in its seed coat. Raw parsley nutritional values per 100 g are as following: carbohydrates 6.3 g, sugars 0.9 g, dietary fiber 3.3 g, fat 0.8 g, protein 3.0 g, vitamins 2.2 mg, calcium 138.0 mg, iron 6.2 mg, magnesium 50.0 mg, phosphorus 58.0 mg, potassium 554 mg, and zinc 1.1 mg (USDA Nutrient Database). Parsley extract inhibits *in vitro *and *ex vivo *platelet aggregation and prolongs bleeding time in rats (Gadi et al., 2009 ▶). Due to its high essential oil content, parsley seeds have a strong diuretic activity (Darias et al., 2001 ▶). Phytochemical screening of parsley has revealed the presence of flavonoids (apiin, luteolin, and apigenin-glycosides) (Fejes et al., 2000 ▶), carotenoids (Francis et al., 1989 ▶), ascorbic acid (Davey et al., 1996 ▶), tocopherol (Fiad et al., 1993 ▶), volatile compounds (myristicin, apiole), coumarines (bergapten, imperatorin) (Fejes, 2000 ▶), phthalides, furanocoumarins, and sesquiterpenes (Spraul et al., 1991 ▶).

 Its recognized roles are: as an antioxidant (Fejes et al., 1998 ▶), anti-inflammatory agent, calcium-channel-blocker in intestine and uterus muscle (Neuhaus-Carlisle et al., 1993 ▶), cancer preventive agent (Zheng et al., 1992 ▶), and also has proved to have laxative (Kreyydieh et al., 2001 ▶), antiulcerogenic (Al-Howiniry et al., 2003 ▶), and hypoglycemic properties (Yanardag et al., 2003 ▶). Moreover, it has a wide reputation as a powerful diuretic in Europe (Tyler, 1993 ▶). 

Prostadin (Prostaglandin F2α) used in this study as an abortifacient is produced by the uterus when stimulated by oxytocin, in the event that there has been no implantation during the follicular phase. It acts on the corpus luteum to cause luteolysis, forming a corpus albicans and stopping the production of progesterone. Action of PGF2α is dependent on the number of receptors on the corpus luteum membrane. Nephropathy means damage to or disease of a kidney. Its causes include IgA antibodies deposition in the glomerulus, administration of analgesics, xanthine oxidase deficiency, and long-term exposure to it or its salts. Chronic conditions that can induce nephropathy include: systemic lupus erythematosus, diabetes mellitus, and high blood pressure (hypertension). Kidney disease is a chronic non-communicable disease and has serious consequences if it cannot be controlled effectively. Generally, the process is from light to serious. Most kidney diseases follow developing process of renal insufficiency, renal failure, and uremia (Giorgino et al., 2004 ▶). Our aim is to investigate the effects of parsley extract on kidney dysfunction caused by abortifacients. Since parsley has been known as a plant with great antioxidant capacity, we used the herb to treat damages caused by the applied drug.

## Materials and Methods

**Collection of plant material**

Seeds of parsley were bought from a medicinal plants store; Naqadeh, West Azerbaijan, Iran. The seeds were identified by Dr. Heidari, A voucher specimen has been kept in herbarium, Urmia University, Urmia (no. 9070). The seeds were powdered and the yield was used for the extract preparation.

**Chemicals**

Prostadin (HP/06/200) was purchased from Nexus Research Ltd, England.

**Preparation of ethanolic extract**

The powdered seed material was macerated with Merck ethanol for 72 h at room temperature using a shaker. Then, it was filtered through Whatmann no.1 filter paper. The solvent was evaporated to dryness using a rotary and the resulted material, a greenish brown solid extract, was powdered and stored in refrigerator for further use (Nawel et al., 2011 ▶). The yield of the extract was ~1.4% (w/w). The crude extract was dissolved in normal saline and administered orally. 

**Induction of abortion**

Abortion was induced in pregnant Wistar albino rats aged 2-3 months (150-180 g body weight) by intraperitoneal injection of prostadin (single dose of 2.295 ml/kg body weight) dissolved in distilled water. All animals were allowed free access to tap water and pellet diet and maintained at room temperature in plastic cages, as per the guidance of Institutional Animal Ethics Committee.

**Experimental design:**

To study the effects of parsley extract, 54 rats were divided into 9 groups each consisting of 6 animals:

1. control (pregnant rats)
2. Parsley (non-pregnant rats)
3. Parsley (pregnant rats)
4. Parsley (first days of pregnancy (DOP))
5. Parsley (second DOP)
6. Parsley (third DOP)
7. Prostadin (non-pregnant rats)
8. Prostadin (pregnant rats)
9. Prostadin + Parsley (Pregnant rats)

Prostadin (dose: 2.295 ml/kg) was administered through intraperitoneal (i.p.) method while parsley extract (5 mg/kg) was given orally. Group’s treatment was administered up to 18 days of pregnancy.

**Metabolic and morphological analysis**

On day 18 of pregnancy, blood samples were obtained directly from heart and biochemical analysis was applied to measure creatinin, urea, malondialdehyde (MDA), and total antioxidant status (TAS).

**Immuno-histochemical and immune-fluorescent staining**

At the end of the treatment period, the kidneys of all animals were separated for histopathological examination. In brief, the kidneys were stored in 10% formaldehyde at room temperature for 24 h, embedded in paraffin, and sectioned (7 μm). Paraffin sections were deparaffinized, hydrated with water, and stained with Hematoxylin-Eosin. Sections were observed under light microscope to determine the number of glomeruli per surface unit, the diameter of bowman capsule, urinary space, and glomeruli count.

**Statistical analysis**

Data were expressed as mean±SEM. The statistical analysis of the difference was carried out using one way analysis of variance (ANOVA) followed by Tukey’s test and significant level was assumed p<0.05.

## Results

**Body weight**

At the end of the treatment, the body weight of treated rats was significantly decreased compared with the control group (Table 1), with parsley-treated group having the highest decrease and lowest decrease observed in DOP groups.

**Assessment of effect of parsley extract on serum parameters**

After 18 days of treatment, the effect of ethanolic extract on serum creatinine, urea, MDA, and TAS in all extract-treated groups was studied. The level of serum creatinine decreased significantly compared with control group in 2^nd^ and 3^rd^ DOP and non-pregnant parsley-treated groups, while other groups showed significant (p<0.05) increase in comparison with control group. Statistical analysis of serum urea showed that there is a significant decrease in pregnant groups while there is a significant increase in non-pregnant groups compared with control group. MDA levels significantly increased in all treated rats compared with those in normal rats, while TAS levels in all experimental groups decreased significantly (p<0.05) (Table 2).

**Table1:** Effects of *Petroselinum crispum *seed ethanolic extract on body weights of normal and treated rats

Group Treatment (n=6)	**Body weight (g) before treatment**	**Body weight (g) after treatment**	**Change in body weight (g)**
Control	150.24±0.08	166.11±5.10	+15.87±5.09
1st DOP+Parsley	169.30±3.02	163.36±5.29	-5.93±2.27*
2d DOP +Parsley	166.26±6.71	164.21±6.18	-2.05±1.96*
3rd DOP +Parsley	159.42±6.63	156.52±5.90	-2.89±1.39*
Pregnant +Parsley	172.91±6.40	143.70±5.73	-29.21±2.43*
Non-pregnant +Parsley	163.76±3.34	158.33±3.17	-5.43±1.60*
Prostadin +pregnant	173.90±4.19	167.03±4.15	-6.86±0.51*
Prostadin +non-pregnant	177.43±2.70	169.46±4.58	-7.96±1.89*
Prostadin +parsley +pregnant	178.73±2.55	155.93±6.71	-22.80±8.95*

* p<0.05. Values are mean±SEM, n=6, when compared with control using one way ANOVA followed by Tukey’s test.

**Table 2:** Effects of *Petroselinum crispum *seed ethanolic extract on serum parameters of normal and treated rats

Group Treatment (n=6)	Creatinine (mg/dl)	Urea (mg/dl)
Control	2.12±0.005	7.82±0.01
1st DOP +Parsley	2.32±0.008*	7.21±0.01**
2d DOP +Parsley	1.63±0.008**	7.32±0.01**
3rd DOP +Parsley	1.83±0.01**	7.21±0.00**
Pregnant +Parsley	2.41±0.005*	7.40±0.005**
Non-pregnant +Parsley	1.94±2.43**	8.32±0.01*
Prostadin +pregnant	2.51±0.008*	7.34±0.008**
Prostadin +non-pregnant	2.25±0.01*	7.94±0.005*
Prostadin +parsley +pregnant	2.41±0.01*	7.40±0.008**

* p<0.05+increase,

** p<0.05+decrease. Values are mean±SEM, n=6, when compared with control using one way ANOVA followed by Tukey’s test.

**Histopathology of kidney**

The study of kidney histology in control animals showed normal structure. In treated rats with prostadin, kidney sections showed mild changes in the density of mesenchyme along with atrophy of glomerular capillaries with increased Bowman's space (urinary space), and severe tubular necrosis (TN). 

**Figure 1:**
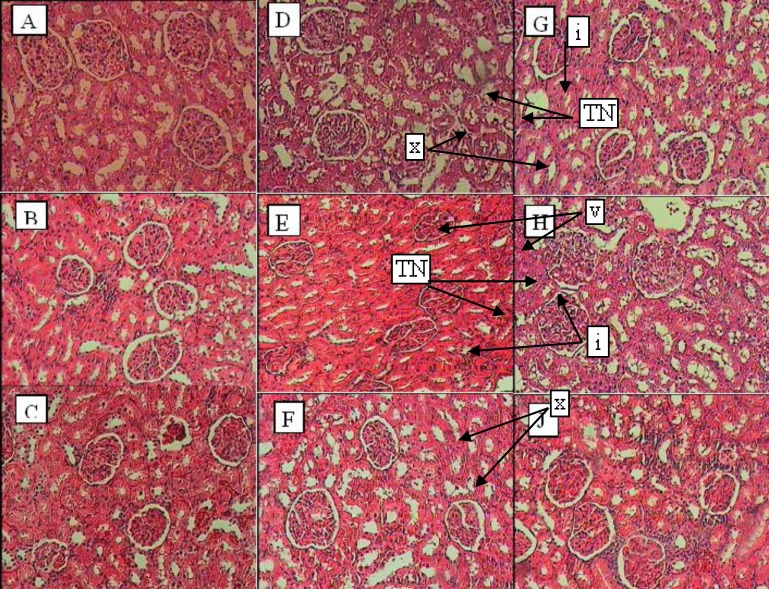
Histopathological evaluation of kidney sections. ×400 magnifition, (A) shows control group, (B) DOP1, (C) DOP2, (D) DOP3, (E) pregnant parsley treatment, (F) parsley non-pregnant treatment, (G) prostadin-treated pregnant group, (H) prostadin-treated non-pregnant group, and (J) mixed treatment group.

The groups that were treated with extract showed features of healing, i.e., normal glomerulus, basement membrane, and capillaries. Moreover, Bowman's space (urinary space) and severe tubular necrosis (TN) were improved towards normal condition after treatment with parsley extract (5 mg/kg) (Figure 1). 

There was no significant difference between control and treated groups in Bowman diameter or glomerular diameter while the difference between DOP3 group with prostadin-treated groups and mixed-treatment group was significant (p<0.05). In the case of glomeruli count, administration of prostadin led to significant decrease, while applying parsley extract increased its number. 

**Table 3:** Effects of *Petroselinum crispum *seed ethanolic extract on MDA and TAS of treated rats

Group Treatment (n=6)	MDA (µmol/L)	TAS (µmol/L)
Control	1.39±0.31	1.25±0.22
1st DOP +Parsley	2.24±0.005*	1.23±0.005**
2d DOP +Parsley	2.23±0.008*	0.95±0.005**
3rd DOP +Parsley	2.20±0.008*	0.92±0.008**
Pregnant +Parsley	3.57±0.008*	0.84±0.008**

* p<0.05+increase,

** p<0.05+decrease. Values are mean±SEM, n=6, when compared with control using one way ANOVA followed by Tukey’s test.

**Table 4:** Effects of *Petroselinum crispum *seed ethanolic extract on bowman and glomerular diameter and urinary space of normal and treated rats

Group treatment (n=6)	**Bowman** **diameter (µm)**	**Glomerular diameter (µm)**	**Urinary space (µm)**	**Glomeruli count (num)**
Control	395±32.84	343±37.00	52±10.05	533.33±51.63
1st DOP +Parsley	351±43.39	298±40.47	53±11.28	666.66±51.63
2d DOP +Parsley	428±53.86	364±51.33	64±6.80	533.33±51.63
3rd DOP +Parsley	467±36.72	396±34.70	71±12.52*	600±89.44
Pregnant +Parsley	377±41.05	329±45.98	48±17.04	733.33±51.63*
Non-pregnant +Parsley	432±40.73	371±46.89	61±16.18	633.33±51.63
Prostadin +pregnant	395±49.04	345±41.61	50±13.76*	333.33±51.63*
Prostadin +non-pregnant	413±29.75	364±29.80	49±8.52*	366.66±51.63
Prostadin +parsley +pregnant	434±43.33	384±43.09	50±9.17*	500±89.44

* p<0.05. Values are mean±SEM, n=6, when compared with control using one way ANOVA followed by Tukey’s test.

**Figure 2:**
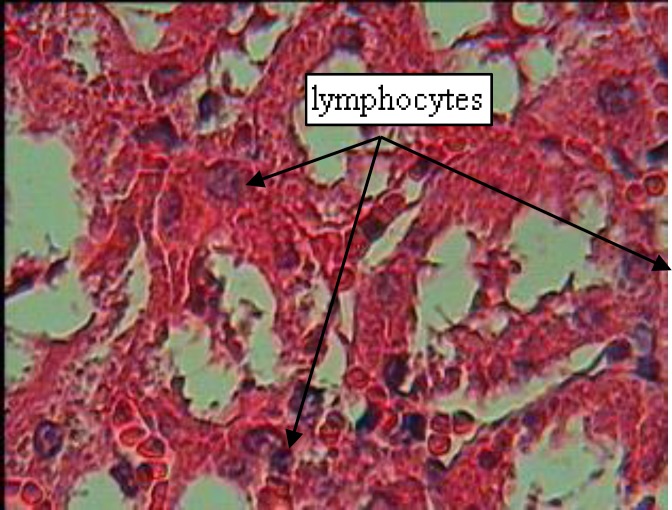
The accumulation of lymphocytes in mixed-treatment group (×400).

## Discussion

 Some functions of synthetic prostaglandins are similar to androgen hormones. But they only perform one of those functions well, which is their anabolic effect and it may lead to not intended complication in body's tissue. 

The PGF2α isoform 8-iso-PGF2α was found in significantly increased amounts in patients with endometriosis, thus it is a potential causative link in endometriosis-associated oxidative stress (Sharma et al., 2010 ▶). Moreover, there are lots of articles discussing that oxidative stress causes major damages to kidney, mostly in diabetes-induced oxidative stresses. However, there are multiple studies about the antioxidant properties of parsley and its effects on oxidative stress-induced tissue damages. Therefore, in this study we investigated the effects of parsley’s antioxidants on renal parameters and damages induced by PGF2α in kidney tissue of rats that had clinical abortion. Our results showed that treatment with both compounds caused weight loss and the shorter the period of administration, the lower the weight lost, which we assume to be due to the lesser period of exposure to extract. Prostadin group showed increase of serum creatinine in both non-pregnant state and pregnancy (post-abortion) in a statically significant manner, while the extract reduced creatinine levels in non-pregnancy and implantation and placentation period but increased significantly in continuous and combined administration and in first DOP period (Alderson et al., 2004 ▶; Mauer et al., 1981 ▶; Ashraf et al., 2013 ▶). 

While in non-pregnant groups which received prostadin or the extract, urea levels increased. It decreased in all of the other groups which indicated that extract-treated rats showed significant improvement in renal functions such as creatinine and urea (Stambe et al., 2003 ▶). Nephropathy reduces the physiological function and changes the structure of kidney (Rosolowsky et al., 2008 ▶). As can be seen in Figure 1, there was significant histopathologic changes in groups treated with prostadin such as severe tubular necrosis or glomerular atrophy, while rats treated with the extract did not show any such signs. The group which was treated with both prostadin and parsley extract showed healing in case of capillary atrophy, changes in mesenchyme density and also lymphocyte accumulation which leads to the presence of an infection in kidney tissue (see Figure 2). 

Antioxidant properties of parsley are likely to be responsible for its effects (Fejes, 2000 ▶). According to literature, parsley is one of the most widely used herbal medicines in Latin America medicine which has been used in clinical settings to treat diabetes and its side effects on kidney and liver for years (Yanardag et al., 2003 ▶). In a study on diabetic rats with nephropathy, parsley extract improved renal parameters and may be useful in the treatment of early stages of human diabetic kidney disease (Sener et al., 2003 ▶).

From the overall results of the biochemical and histopathological examinations, it could be inferred that ethanolic extract of *Petroselinum crispum* possesses beneficial effects on kidney parameters in abortion-induced kidney dysfunction.

## Conflict of interest

There is not any clash of attentiveness in this study.
